# Increased expression of Toll-like receptors 2, 3, 4 and 7 mRNA in the kidney and intestine of a septic mouse model

**DOI:** 10.1038/s41598-019-40537-2

**Published:** 2019-03-08

**Authors:** Sylvia Krivan, Alkistis Kapelouzou, Stylianos Vagios, Diamantis I. Tsilimigras, Michalis Katsimpoulas, Demetrios Moris, Chrysostomos V. Aravanis, Theano D. Demesticha, Dimitrios Schizas, Manolis Mavroidis, Kitty Pavlakis, Anastasios Machairas, Evangelos Misiakos, Theodore Liakakos

**Affiliations:** 1 Department of Upper Gastrointestinal and Bariatric Surgery, Luton and Dunstable University Hospital, LU4 0DZ Luton, United Kingdom; 2 Center for Clinical, Experimental Surgery and Translational Research, Biomedical Research Foundation of the Academy of Athens, 11527 Athens, Greece; 3 School of Medicine, National and Kapodistrian University of Athens, 11527 Athens, Greece; 4 First Department of Surgery, Laikon General Hospital, National and Kapodistrian University of Athens, 11527 Athens, Greece; 5 Department of Anatomy, Faculty of Medicine, National and Kapodistrian, University of Athens, 11527 Athens, Greece; 6 Department of Pathology, Medical School, National and Kapodistrian University of Athens, 11527 Athens, Greece; 7 Third Department of Surgery, Attikon General Hospital, Medical School, National and Kapodistrian University of Athens, 11527 Athens, Greece

## Abstract

Toll-like receptors (TLRs) are the key regulators of innate and adaptive immunity and are highly expressed during sepsis. Thus, studying the expression of TLRs in an animal septic model might indicate their possible association with acute kidney injury in sepsis. Seventy-two male C57BL/6J mice were used for this study. Randomly, these animals were divided into 6 groups (N = 12/group): 3 control and 3 septic groups depending on the euthanasia time (24 h, 48 h, 72 h). Septic groups underwent cecal ligation and puncture (CLP) to induce peritonitis, while control groups had a sham operation. Hematological tests were performed in serum for immune biomarkers; immunohistochemistry, morphometry and qRT-PCR analysis were used on both kidney and intestine tissues to evaluate the expression of TLR 2, 3, 4 and 7 in a septic process. At the end of each experimental period, we found that TLRs 2, 3, 4 and 7 were expressed in both tissues but there were differences between those at various time points. Also, we found that mRNA levels were significantly higher in qRT-PCR evaluation in septic groups than control groups in both kidney and intestinal tissues (p < 0.05); showing a steady increase in the septic groups as the time to euthanasia was prolonged (p < 0.05). Overall, our study provides a suggestion that TLRs 2, 3, 4 and 7 are highly expressed in the kidneys of septic mice and especially that these TLRs are sensitive and specific markers for sepsis. Finally, our study supports the diagnostic importance of TLRs in AKI and provides an insight on the contribution of septic mice models in the study of multi organ dysfunction syndrome in general.

## Introduction

Sepsis is defined as a systemic, dysregulated immunologic host response to infection which can result in multiple organ dysfunction syndrome (MODS) and, often, it is incompatible with life^1,2^. It remains the primary cause of death in intensive care unit (ICU) patients^3^. Several different mediators, such as cytokines, chemokines, complement-activating products and Toll-like receptors (TLRs), have been recognized to be involved in the pathogenesis of sepsis, each serving in independent or common pathways. However, the intricacy of the liable mechanisms has made it difficult to understand their exact nature^4,5^. On these grounds, animal septic models have been used extensively, so far, to reproduce the complexity of human sepsis. One of the most frequently used models is cecal ligation and puncture (CLP) which causes peritonitis and, subsequently, sepsis through polymicrobial infection in a way that resembles the human response^6–8^. Toll-like receptors (TLRs) are germ-line-encoded type I transmembrane proteins expressed in various immune as well as non-immune cells and belong to a family of pattern recognition receptors (PRPs)^9^. TLRs recognize and are activated by certain pathogen-associated molecular patterns (PAMPs)^10^, such as lipopolysaccharides, lipoproteins, peptidoglycans) leading to stimulation of the innate immune system and subsequently to activation of antigen-specific adaptive immunity^11^. The characterization and expression of these receptors is, therefore, essential in understanding the pathophysiology of sepsis and more specifically the related organ dysfunction^12^.

Sepsis is one of the most common causes of acute kidney injury (AKI)^13^. For many years, it was thought that sepsis-related hemodynamic alterations in the macrocirculation resulting in reduced renal perfusion were responsible for this phenomenon. This mechanism has been outdated, and a more complex pathway is suggested; changes in the microcirculation of the kidney along with an exacerbated inflammatory response propose a more accurate, although not completely clarified, theory^14^. This strongly suggests the presence of a common pathway between the initial triggers of tubular cell injury and the inflammatory response in the kidney^15,16^.

Within the kidney different cell types express some of the TLR system proteins. In bacterial infections affecting the kidney, upregulation of TLRs -2, -3 and -4 and subsequent C-C chemokines secretion has been described^17^. Thus, TLR activation may be the common denominator amongst various forms of tubular cell injury and, more specifically, the trigger of the “innate” immune response leading to AKI in a septic state, such as during a CLP mouse model^13,18^.

In the present study, we examined the pathogenic mechanism of AKI in relation to TLR expression. Thus, we used a septic mouse model which is representative of a clinical patient’s situation^19^ to determine the role of TLRs 2, 3, 4 and 7 in the severity of sepsis, as well as its association with multi-organ dysfunction syndrome triggered by AKI.

## Materials and Methods

### Animal study and care

Seventy-two male C57BL/6J mice, aged 12–14 weeks and weighing 20–25 g supplied from the colony of the Center of Experimental Surgery at our Institute were sacrificed. This study protocol was approved by the local ethics committee (Athens Prefecture Veterinarian Service; 4854/27-07-2012; code EL 25 BIO 003). All experiments took place in the animal facilities of the Center of Experimental Surgery, Biomedical Research Foundation, Academy of Athens (BRFAA) according to the guidelines set by the National Research Council’s Guide for Care and Use of Laboratory Animals.

### Experimental Design and establishment of CLP Procedure

A clinically relevant mice model of sepsis was created by cecal ligation and puncture (CLP). The protocol of the study has previously been published and the samples referenced in this study derive from the same 60 animals used in the study by Bakopoulos *et al*.^20^ with the addition of 12 new animals (2 new animals/group/each time table). The control group mice underwent a sham surgery receiving a laparotomy without cecal ligation and puncture. All animals were resuscitated with 1 mL isotonic sodium chloride solution administered subcutaneously. The mice of the septic and the control groups were sacrificed 24, 48 and 72 hours following the CLP procedure and the sham operation, respectively, forming 6 groups; 24S, 48S and 72S representing the septic groups, and 24C, 48C, 72C the control ones. At the end of the experimental period for each group, euthanasia was performed. Part of each tissue obtained was formalin fixed and paraffin embedded for histological analysis; the rest was rinsed with distilled (DEPC-treated) water and shock frozen to −140 °C for mRNA analysis. The experimenters were not blind to the surgical procedure but they were blind to all the other experimental analysis.

### Measurement of serum biomarkers and inflammatory mediators

Different serum biomarkers were measured to evaluate the kidney injury after CLP. More specifically, blood samples were drawn with intra-cardiac aspiration after each experimental period. Those samples were used for the assessment of several biochemical and immunological markers to evaluate the kidney injury. Serum urea, creatinine, total and direct bilirubin were measured in Chemical Awareness analyzer (Awareness Tech, USA) with the corresponding commercial kit (Human, Wiesbaden, Germany). Elisa kits were used to determine mouse serum interleukin 18 (IL18) (MBL International, Woburn, USA) and neutrophil gelatinase associated-lipocalin (NGAL) (R&D systems, Abingdon, UK) according to the manufacturers’ instructions.

### Histological examination

Five μm serial paraffin kidney sections collected from all experimental animals were cleared in xylene and hydrated with different grades of absolute alcohols. Subsequently, those renal sections were hematoxylin and eosin stained for the study and evaluation of tissue injury.

### Kidney immunofluorescence staining of TLR 2, 3, 4 and 7

Immunofluorescence staining was performed for the following primary polyclonal rabbit TLR -2 (1:200 dilution; sc10739; Santa Cruz Biotechnology, INC), TLR -3 (1:200 dilution; sc28999; Santa Cruz Biotechnology, INC), TLR -4 ((1:200 dilution; sc30002; Santa Cruz Biotechnology, INC) and TLR -7 ((1:200 dilution; sc30004; Santa Cruz Biotechnology, INC). A goat anti-rabbit antibody TRITC- ((1:200 dilution; sc2780; Santa Cruz Biotechnology, INC) was used as a secondary antibody. All images were acquired using microscope Leica DM RXA2 and DM RA2 (Leica Microsystems Wetzlar GmbH, Germany) with Hamamatsu ORCA-Flash 4.0 V2 camera (Hamamatsu Photonics Deutschland GmbH, Germany).

### Morphometrical analysis

Morphometrical analysis was performed in immunofluorescence staining sections by densitometry, to estimate: (a) the expression fraction of kidney; (b) the percentage of the cellular nuclei; (c) the percentage of each TLR expression, using the Image J Program (version 1.49C, Wayne Rasband, National Institute of Health, USA) to analyze every photo.

### Real-Time Polymerase Chain Reaction (PCR)

Total RNA was extracted with TRI reagent (Life Technologies-Invitrogen) from kidney and intestine tissue samples according to the manufacturer’s protocol^21^. Before being reversed into cDNA, RNA samples were treated with DNase I recombinant, RNase-free (Roche, Mannheim Germany) 1U/μg DNA plus 40U RNaseOut Ribonuclease Inhibitor (Invitrogen) incubated at 37 °C for 35 min. Samples were heat inactivated and extracted with phenol/chloroform. 1 μg total renal or intestine RNA was reversed into cDNA. mRNA analysis of TLRs 2, 3, 4 and 7 was performed using Power SYBR Green Master Mix (Applied Biosystems, UK) on a LightCycler 480 (Roche Mannheim, Germany). Integrated DNA Technologies (Leuven, Belgium) provided all primers such as (a) TLR 2 (sense:5′-tgggctgacttctctcaatg-3′, antisense:5′-ttcatcggtgagctgacttc-3′); (b) TLR 3 (sense:5′-ctgggtctgggaacatttct-3′, antisense:5′-ttgctgaactgcgtgatgta-3′); (c) TLR 4 (sense:5′-ggaacaaacagcctgagaca-3′, antisense:5′-ttgagactggtcaagccaag-3′); (d) TLR 7 (sense:5′-atacctggccactgatgtga-3′, antisense:5′-gactccatggattgcagatg-3′); and (e) GAPDH (sense:5′-ccagaatgaggatcccagaa-3′,antisense:5′-accacctgaaacatgcaaca-3′). All experiments were repeated 3 times. The relative changes in gene expression were assessed with the use of the 2-ΔΔCT analysis method^22^. All data from relative level mRNA expression for each gene in each sample were normalized by GAPDH levels.

### Statistical Analysis

The assumption of normality in our data was checked using the Shapiro-Wilk test. Differences in blood test results and TLR expression between the septic and control groups were assessed with one-way Anova followed by Tukey’s test analysis (mean ± SD). Every difference with p < 0.05 was considered of statistical significance. All statistical analysis was performed using Graph Pad Prism v 4.03 for Windows.

## Results

### Serum biomarkers during experimental sepsis

Renal dysfunction is an established side effect of sepsis secondary to peritonitis, among other organs such as the lungs and the liver. To evaluate the onset and extent of AKI, several blood serum biomarkers were investigated in our study. Standard parameters, such as urea and creatinine, although slightly outdated but still reliable^23^ were measured in both control and septic groups at different time points. During the experimental period, statistical differences were found between CLP and sham groups. There was a significant increase in each serum marker highlighted among the septic groups, in a time dependent manner, with statistical significance being more profound between S24 and S72 groups (p < 0.05) (Table 1). Moreover, to investigate renal inflammation, NGAL and IL18 markers were measured and their serum levels were monitored after CLP and sham operation. IL18 and NGAL also rose similarly as blood biomarkers to a peak at 72 hours after CLP (Table 1), proving as potentially related biomarkers for sepsis-induced AKI^24^.

**Table 1:** Serum concentrations of biomarkers.

Time Biochemical markers	24 h	48 h	72 h
**Urea (mg/dL)**			
Control	39.25 ± 10.26^a^	48.50 ± 11.67^a^	54.33 ± 9.921^a^
Septic	77.58 ± 12.87	137.1 ± 23.92^b^	237.8 ± 45.97^c,d^
**Creatinine (mg/dL)**			
Control	0.372 ± 0.157^a^	0.478 ± 0.149^a^	0.45 ± 0.16^a^
Septic	1.502 ± 0.24	2.473 ± 0.287^b^	4.414 ± 0.825^c,d^
**Total Bilirubin (U/L)**			
Control	0.779 ± 0.15^a^	0.729 ± 0.167^a^	0.674 ± 0.21^a^
Septic	1.846 ± 0.378	3.648 ± 0.273^b^	6.82 ± 0.83^c,d^
**Direct Bilirubin (mg/dL)**			
Control	0.676 ± 0.106^a^	0.655 ± 0.104^a^	0.64 ± 0.084^a^
Septic	1.128 ± 0.1052	1.603 ± 0.16^b^	3.142 ± 0.473^c,d^
**IL-18 (pg/ml)**			
Control	44.94 ± 9.769^a^	47.62 ± 6.89^a^	42.22 ± 14.18^a^
Septic	167.9 ± 21.63	263.1 ± 25.99^b^	354.9 ± 32.94^c,d^
**NGAL (pg/ml)**			
Control	5.628 ± 1.173^a^	6.145 ± 1.745^a^	6.113 ± 0.815^a^
Septic	173 ± 19.77	255.4 ± 21.36^b^	570.1 ± 39.32^c,d^

Values are expressed as mean ± SD. N = 12 for each experimental group. Statistical significances (p < 0.05) between the groups at the same time point are indicated as follows: ^a^Control vs Septic; ^b^S24 vs S48; ^c^S48 vs S72; ^d^S24 vs S72.

### Renal histopathology and Immunofluorescence

Toll-like receptors (TLRs) are the first identified family of pattern recognition receptors. They have been best studied and it is common knowledge that they are expressed in immunocytes, as well as widely distributed in various cell types, including renal cells. Hence, they contribute significantly to various pathologies of the kidney^25^. They respond to remote signals and increase their expression, evidence found in both histopathology and immunofluorescence.

**Figure 1:**
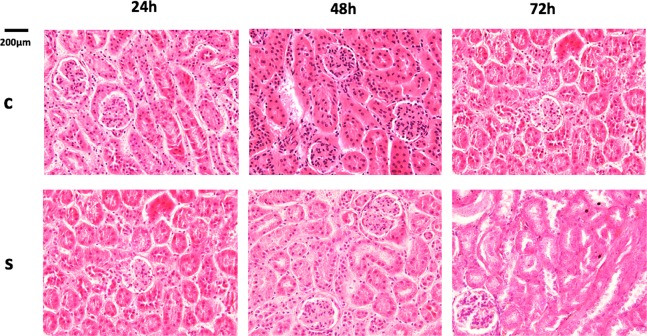
Representative photos from the severity of renal injury after CLP procedure during various times. Tubular dilation increased during time. Original magnification 40x; 200 μm.

**Figure 2:**
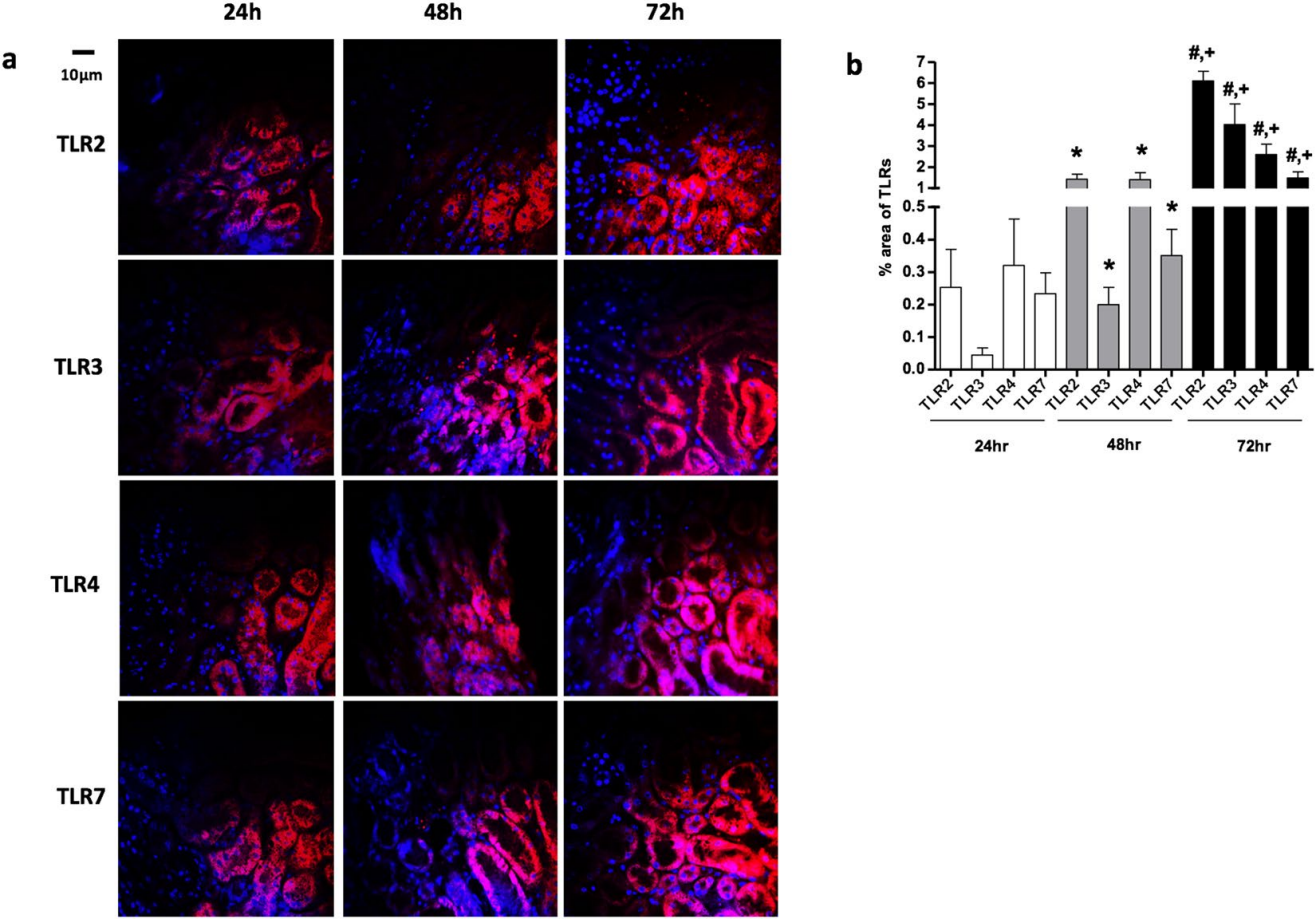
(**a**) Representative images of immunofluorescence analysis in the septic groups kidney tissues concerning TLRs 2, 3, 4 and 7 at all time points. Red color shows antibodies expression; Blue color shows DAPI stain for nucleus. (**b)** Immunofluorescence analysis proved a gradual increase in the expression of TLRs 2, 3, 4 and 7 in the kidney tissues of the septic mice. This observation was statistically significant concerning all TLRs at all time-point progressions. Statistical significances (p < 0.05) between different time points are indicated as follows: ^*^S24 vs S48; ^#^S24 vs S72; ^+^S48 vs S72.

In our study, as far as histological changes are concerned, they were more prominent in tubular dilation in septic groups rather than in the control groups; additionally, more extensive renal damage was observed in a time dependent manner in septic groups (Fig. 1). Furthermore, immunofluorescence analysis revealed that cells around kidney tubules expressed TLR 2, 3, 4 and 7 in all septic groups (Fig. 2a). As indicated in our experimental data results (Table 2), there was a significant increase of TLR 2, 3, 4 and 7 in septic mice over time (Fig. 2b) (p < 0.05). In contrast, control mice demonstrated no detectable expression of these TLRs.

**Table 2:** Immunofluorescence quantification of kidney tissues.

Time Points Area (%) of expression, of TLRs	24 h	48 h	72 h
**TLR-2**			
Control	nd	nd	nd
Septic	0.253 ± 0.117	1.423 ± 0.247^a^	6.1 ± 0.465^b,c^
**TLR-3**			
Control	nd	nd	nd^a^
Septic	0.045 ± 0.022	0.2 ± 0.053^a^	4.026 ± 0.983^b,c^
**TLR-4**			
Control	nd	nd	nd
Septic	0.320 ± 0.143	1.407 ± 0.331^a^	2.598 ± 0.492^b,c^
**TLR-7**			
Control	nd	nd	nd^a^
Septic	0.234 ± 0.064	0.351 ± 0.08^a^	1.49 ± 0.29^b,c^

Values are expressed as mean ± SD. N = 12 for each experimental group. Statistical significances (p < 0.05) between the groups at the same time point are indicated as follows: ^a^S24 vs S48; ^b^S48 vs S72; ^c^S24 vs S72; nd: non detectable.

### CLP enhances TLR signaling molecule expression in kidney and intestine tissue

**Figure 3:**
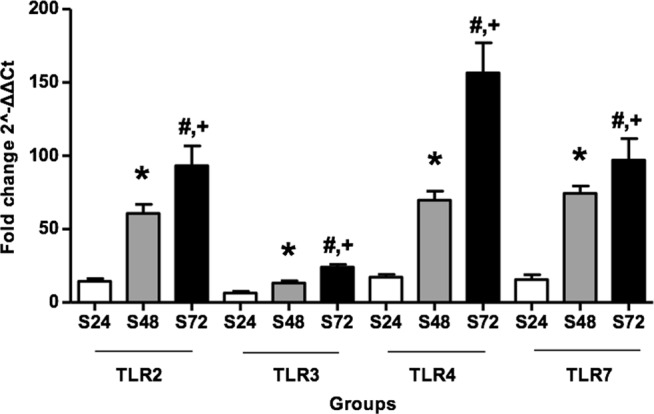
Quantitative real time polymerase chain reaction analysis of TLRs 2, 3, 4 and 7 mRNA expression in kidney tissues after CLP. Data are normalized to housekeeping gene GADPH expression. Values are mean ± SD; 12 animals per group. Statistical significances (p < 0.05) between different time points are indicated as follows: ^*^S24 vs S48; ^#^S24 vs S72; ^+^S48 vs S72.

**Figure 4:**
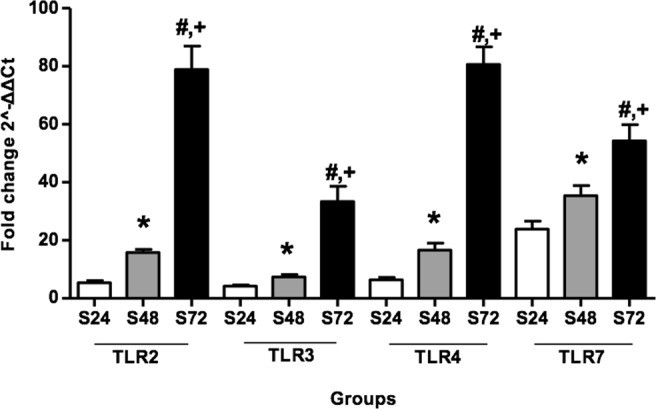
Relative mRNA expression of TLRs 2, 3, 4 and 7 estimated by quantitative polymerase chain reaction in intestine tissues after CLP. Data are normalized to housekeeping gene GADPH expression. Values are mean ± SD; 12 animals per group. Statistical significances (p < 0.05) between different time points are indicated as follows: ^*^S24 vs S48; ^#^S24 vs S72; ^+^S48 vs S72.

The expression of TLR 2, 3, 4 and 7 receptor genes was quantified by RT-PCR after 24, 48 and 72 hours of CLP induction in both, kidneys (Table 3) and intestine (Table 4). Each time point in the graphs (Fig. 3; kidney and Fig. 4; intestine) represents the fold change in expression, resulting from septic groups being compared with control groups, using the 2^−ΔΔCT^ method. No significant changes in expression were demonstrated between control groups at any time point (data not shown). On the contrary, increased levels of TLR expression were observed after 72 hours of CLP induction in both kidney and intestine tissues. A statistically significant increase concerning each TLR was demonstrated at the prolonged septic process (p < 0.05).

**Table 3:** mRNA expression of Toll like Receptors in renal tissues.

Time Points TLRs ratio Septic/Control	24 h	48 h	72 h
TLR-2	14.48 ± 1.758^a^	60.74 ± 6.097^a,b^	93.21 ± 13.57^a–d^
TLR-3	6.476 ± 1.135^a^	13.26 ± 1.497^a,b^	24.00 ± 1.863^a–d^
TLR-4	17.25 ± 1.841^a^	69.68 ± 6.154^a,b^	156.5 ± 20.54^a–d^
TLR-7	15.57 ± 3.275^a^	74.40 ± 5.000^a,b^	97.10 ± 14.66^a–d^

Values are expressed as mean ± SD. N = 12 for each experimental group. Statistical significances (p < 0.05) between the groups at the same time point are indicated as follows: ^a^Control vs Septic; ^b^S24 vs S48; ^c^S48 vs S72; ^d^S24 vs S72.

**Table 4:** mRNA expression of Toll like Receptors in intestinal tissue.

Time Points TLRs ratio Septic/Control	24 h	48 h	72 h
TLR-2	5.414 ± 0.705^a^	15.83 ± 1.061^a,b^	78.87 ± 8.051^a–d^
TLR-3	4.216 ± 0.439^a^	7.364 ± 0.885^a,b^	33.38 ± 5.239^a,c,d^
TLR-4	6.383 ± 0.862^a^	16.62 ± 2.414^a,b^	80.64 ± 6.059^a–d^
TLR-7	23.85 ± 2.8^a^	35.33 ± 3.556^a,b^	54.32 ± 5.515^a–d^

Values are expressed as mean ± SD. N = 12 for each experimental group. Statistical significances (p < 0.05) between the groups at the same time point are indicated as follows: ^a^Control vs Septic; ^b^S24 vs S48; ^c^S48 vs S72; ^d^S24 vs S72.

**Figure 5:**
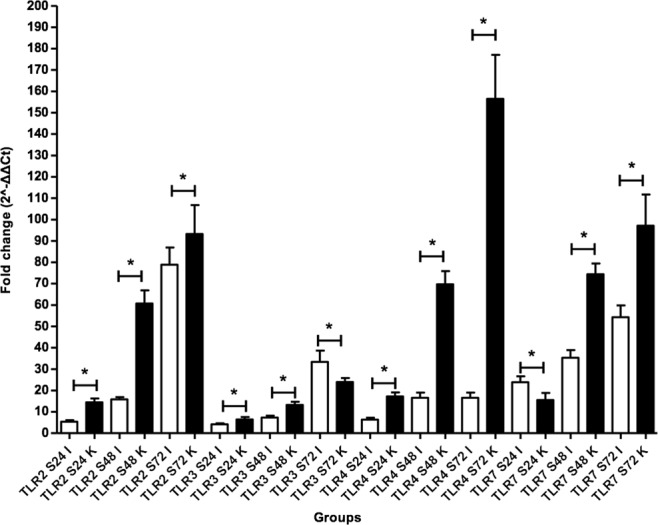
Differential expression of TLRs in the kidneys contrary to the intestine in the septic groups. Statistical significances (p < 0.05) between intestine and kidney are shown as *.

In Fig. 5, the difference of each TLR expression between the kidney and the intestine tissues of the septic mice is depicted. Comparisons were made between septic groups of the respective euthanasia time. TLR 2 and TLR 4 gene expression was found higher in the kidney compared to intestine tissue during sepsis. At 72 h, the levels of the TLR 3 gene were significantly higher in intestine compared to kidney tissue. In contrast, at 24 h and 48 h TLR 3 gene expression was higher in the kidney tissue. Last, but not least, at 24 h sepsis, TLR 7 gene expression was significantly higher in the intestine tissue.

## Discussion

Sepsis, a commonly encountered complication in an intensive care unit (ICU), often leads to multi-organ dysfunction and the kidney is one of the organs frequently afflicted. Despite considerable research during the last decades, the pathophysiology of sepsis-induced-AKI remains incompletely understood.

In the present model of sepsis induced AKI (CLP septic model), serum creatinine, urea, total and direct bilirubin, NGAL and IL18 levels were significantly increased during the experimental period. This phenotype of renal injury is acting in the same way with AKI in patients^26^.

Our study followed the same concept, principle and protocol to two other already published articles in literature. Aravanis *et al*. studied the expression of liver function tests (LFTs) and the TLR expression in the hepatic and intestinal tissues after CLP induced peritonitis^27^, whereas Bakopoulos *et al*. concentrated on the expression of hematological and biochemical markers related to respiratory failure as well as TLR expression in the lung and intestine after the same experimental procedure^20^. We, on the other hand, measured AKI biochemical and specific immune biomarkers, in addition to TLR expression in the kidney and the intestine after CLP. Liver function tests, AKI biochemical and immune biomarkers as well as respiratory biochemical markers and monocytes were increased similarly in septic groups versus control groups in a time dependent manner. On the contrary, hematological markers were decreased in the septic groups of sepsis-induced ARDS gradually over time. All TLRs displayed increased expression in septic versus control groups over time in all remote organs (liver, lung, kidney) secondary to CLP induced intra-abdominal peritonitis. TLRs have been found to be associated with sepsis-related dysfunction of various organs such as the intestine, the lung and the liver^20,28^. Analysis of TLR 2, 3, 4 and 7 expression in the intestine tissues of septic mice groups, showed a statistically significant increase in each TLR expression, progressively from 24 h to 72 h euthanasia time. Comparing control groups with time equivalent septic mice produced statistically significant differences for every TLR in each different euthanasia time.

The expression of TLR 2, 3, 4 and 7 was also found to be upregulated in the kidneys of septic mice, whilst it was only negligible in the kidney of control groups. These results were of statistical significance for each single TLR between time-equivalent groups (S24 vs C24, S48 vs C48, S72 vs C72) in qRT-PCR analysis. This data also suggests that, potentially, bacteria released from an injured intestine mediate inflammatory responses via TLR 2, 3, 4 and 7 within a different organ than the gut, such as the kidney. Destruction of Gram-negative bacteria results in the release of LPS into the bloodstream where binds to the LPS-binding protein (LBP). The LPS-LBP complex could bind to monocytes, macrophages and neutrophils^29^, through the co-receptor CD14 and interactions with the cell surface Toll-like receptor 4-MD-2 complex. This complex also binds to other cells, including renal tubular epithelial cells^30^, which are then stimulated to produce cytokines through a myeloid differentiation primary response gene (MyD88)-dependent and an MyD88-independent pathway^31^. The septic AKI has been shown to be dependent mainly on MyD88b^32^.

In the present study, a CLP septic model was used to examine the progression of sepsis, since it remains the most reliable animal model to resemble human conditions^6^. Various biomarkers expressed in organ failure were analyzed to examine the effect of TLR activation and expression in sepsis and eventually, MODS.

Additionally, serum NGAL as well as IL18 were similarly increased to the other serum biomarkers. This highlights the fact that systemic inflammation responds to high levels of NGAL. Previous studies suggest that neutrophil gelatinase associated lipocalin (NGAL) activity is a very sensitive marker of AKI, and it is produced by injured tubular epithelial cells^33,34^. Alteration of NGAL serum levels expressed in patients with AKI, identify the organ pathology^35,36^. Overall from our study we show that targeting NGAL levels provides kidney injury during sepsis and is associated with the inflammatory stress that is enhanced in the presence of IL18, the critical mediator of sepsis.

There are two phases of immune responses secondary to infection involved in the pathogenesis of sepsis^37,38^- both of which are displayed in a CLP model: systemic inflammatory response (SIRS) and compensatory anti-inflammatory response (CARS). Acute-phase proteins (CRP, procalcitonin), as well as pro-inflammatory cytokines (TNF*α*, IL1*β*, IL6, IL18) are common biomarkers for SIRS and significantly increase early in the onset of the septic response^39,40^. Single or multiple organ failure may be the result of the “cytokine” storm, which refers to these pro-inflammatory cytokines reaching their highest levels^41,42^. The second phase of the immune response is mediated by the secretion of anti-inflammatory cytokines (IL-4, IL-10). A moderate increase of these cytokines in the blood stream can play a protective role against the organ dysfunction and subsequently promote an antimicrobial immune response^43^. Thus, it is important to study and measure all mediators participating in this cascade and a serum cytokine profile could be used as a prognostic biomarker to predict the severity of the disease.

Furthermore, current studies suggest the potential role of IL18 is multifactorial including increases in septic mice during time. IL18 is an important pro inflammatory cytokine found in intestinal and immune system cells^44^. Circulating IL18 increases during sepsis and is strongly correlated with the severity of the disease^45^. Currently, our results enhance those previous studies. We observed elevation of the circulating IL18 in this CLP model in septic groups at all experimental points, indicating involvement in organ failure, since it is believed that high levels of IL18 are harmful in sepsis. More specifically, IL18 increased dramatically in S24 and S48 groups, representing an early onset of the cytokine storm in the setting of sepsis.

Also, biochemical markers are associated with the alterations in IL18 levels during sepsis. Renal function is mainly monitored by serum creatinine and secondarily urea, the levels of which along with the urine output and other markers define AKI^46^. We analyzed the differences in the expression of both creatinine and urea in septic and control mice at all euthanasia times (24 h, 48 h and 72 h). These two markers were at normal and unchanging levels in the control groups. On the other hand, there was a constant and significant increase among the septic groups as the time of euthanasia was prolonged, which also lead to significant differences between the time-adjusted control and septic groups. In our results, an alike pattern of expression alterations was denoted for total and direct bilirubin, as well, indicating a possible dysfunction of other organs such as the liver or the lung^47^.

So far, eleven human and thirteen mouse TLRs have been identified. These type I transmembrane proteins recognize pathogen-associated molecular patterns and trigger immune responses and inflammatory cascades^9^. Not all TLRs act upon the same targets; TLR-2 recognizes gram (+) bacteria, TLR-4 responds to gram (-) bacteria, whilst TLRs -3 and -7 counter viruses^48^. TLRs -1, -2, -4, -6 are expressed in interstitial and glomerular macrophages; tubular cells and mesangial cells express the same proteins with the addition of TLR 3, while dendritic cells within the kidney express TLR -4, -7, -8 and -9^49^. The upregulation of these TLRs in AKI of septic etiology, which for some TLRs is shown in other literature studies as well, might describe the involvement of these particular proteins in this pathology^13,49,50^.

One of the interesting results of our study is the significant difference in TLR 7 expression levels in kidney tissues of all septic groups when compared to the control ones and the statistically significant increase among the septic groups as the euthanasia time is prolonged in every single comparison in PCR analysis. The role of TLR 7 in the pathogenesis of sepsis is yet to be proven, however, our results suggest that more studies should address to this direction, with TLR 7 being one of the least studied TLRs in sepsis-induced kidney injury. It appears that TLRs are involved in the initiation of the septic cascade and their levels seem to be associated with the severity of sepsis and its progression to organ dysfunction.

## Conclusion

TLR2,3,4 and 7 have a role in sepsis-induced AKI. Further studies should be undertaken to map the exact pathways of sepsis development and the exact role of TLRs during this process aiming to possibly alter the disease course by modulating these proteins.
